# A Randomized Controlled Trial of Interventions to Impede Date Palm Sap Contamination by Bats to Prevent Nipah Virus Transmission in Bangladesh

**DOI:** 10.1371/journal.pone.0042689

**Published:** 2012-08-08

**Authors:** Salah Uddin Khan, Emily S. Gurley, M. Jahangir Hossain, Nazmun Nahar, M. A. Yushuf Sharker, Stephen P. Luby

**Affiliations:** 1 International Centre for Diarrhoeal Disease Research, Bangladesh (ICDDR,B), Dhaka, Bangladesh; 2 Centers for Disease Control and Prevention (CDC), Atlanta, Georgia, United States of America; The University of Hong Kong, China

## Abstract

**Background:**

Drinking raw date palm sap is a risk factor for human Nipah virus (NiV) infection. Fruit bats, the natural reservoir of NiV, commonly contaminate raw sap with saliva by licking date palm’s sap producing surface. We evaluated four types of physical barriers that may prevent bats from contacting sap.

**Methods:**

During 2009, we used a crossover design and randomly selected 20 date palm sap producing trees and observed each tree for 2 nights: one night with a bamboo skirt intervention applied and one night without the intervention. During 2010, we selected 120 trees and randomly assigned four types of interventions to 15 trees each: bamboo, *dhoincha* (local plant), jute stick and polythene skirts covering the shaved part, sap stream, tap and collection pot. We enrolled the remaining 60 trees as controls. We used motion sensor activated infrared cameras to examine bat contact with sap.

**Results:**

During 2009 bats contacted date palm sap in 85% of observation nights when no intervention was used compared with 35% of nights when the intervention was used [p<0.001]. Bats were able to contact the sap when the skirt did not entirely cover the sap producing surface. Therefore, in 2010 we requested the sap harvesters to use larger skirts. During 2010 bats contacted date palm sap [2% vs. 83%, p<0.001] less frequently in trees protected with skirts compared to control trees. No bats contacted sap in trees with bamboo (p<0.001 compared to control), *dhoincha* skirt (p<0.001) or polythene covering (p<0.001), but bats did contact sap during one night (7%) with the jute stick skirt (p<0.001).

**Conclusion:**

Bamboo, *dhoincha*, jute stick and polythene skirts covering the sap producing areas of a tree effectively prevented bat-sap contact. Community interventions should promote applying these skirts to prevent occasional Nipah spillovers to human.

## Introduction

**Figure 1:**
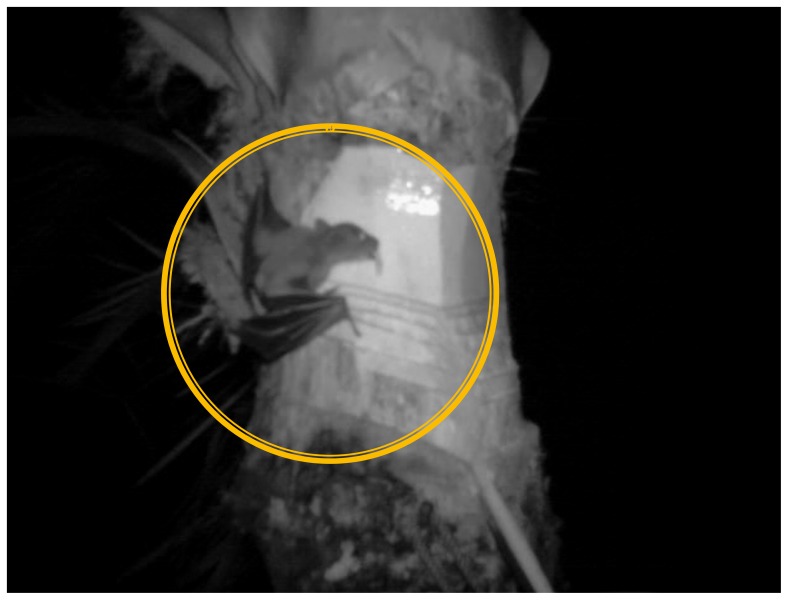
A picture taken by infrared night observation showing a small fruit bat (in circle) licking sap from the shaved surface of a date palm tree without any intervention during the winter of 2010.

Nipah virus (NiV) causes seasonal outbreaks in humans in Bangladesh that coincide with the date palm sap harvesting season, November to March [1]. The first human outbreak was detected in Bangladesh in 2001. Up to 2010 investigators have identified 10 Nipah outbreaks with a total of 173 cases, 131 (76%) of whom died [2], [3]. Fruit bats, the apparent natural reservoirs of NiV, occasionally shed the virus in saliva and urine [4]–[7]. During outbreak investigations, drinking raw date palm sap was identified as a risk factor for NiV infection in humans [8]–[11]. Researchers also identified fruit bats frequently visiting date palm trees and licking the sap [Figure 1] [12]. Although dropped fruit has occasionally been posited as a pathway for transmission, it has never been associated with human NiV infection in outbreak investigations in Bangladesh [2]. Similarly, while there is some evidence of occasional transmission through domestic animals, this pathway represents a much less important route of transmission in Bangladesh compared with date palm sap [2]. Preventing the original spillover of Nipah from bats to people through date palm sap can also prevent subsequent cases of person-to-person transmission of NiV which has been repeatedly observed in Bangladesh [13], [14].

Exploratory studies on date palm sap production and harvesting processes identified several interventions practiced by the sap harvesters to prevent bats feeding on date palm sap [15]. The most promising of these interventions were skirts of various materials including bamboo, *dhoincha* (a local plant; *Sesbania aculeata*), jute sticks (*Corchorus spp*.) or polythene that created a physical barrier to limit bat access to date palm sap during collection. Subsequent small scale intervention trials suggest that date palm sap collectors were willing to make and try the skirts [16].

To evaluate the efficacy of these interventions to protect sap from bats, we conducted randomized controlled trials of the locally identified interventions that formed a physical barrier on the sap producing surface of date palm trees.

## Methods

### Study Settings

**Figure 2:**
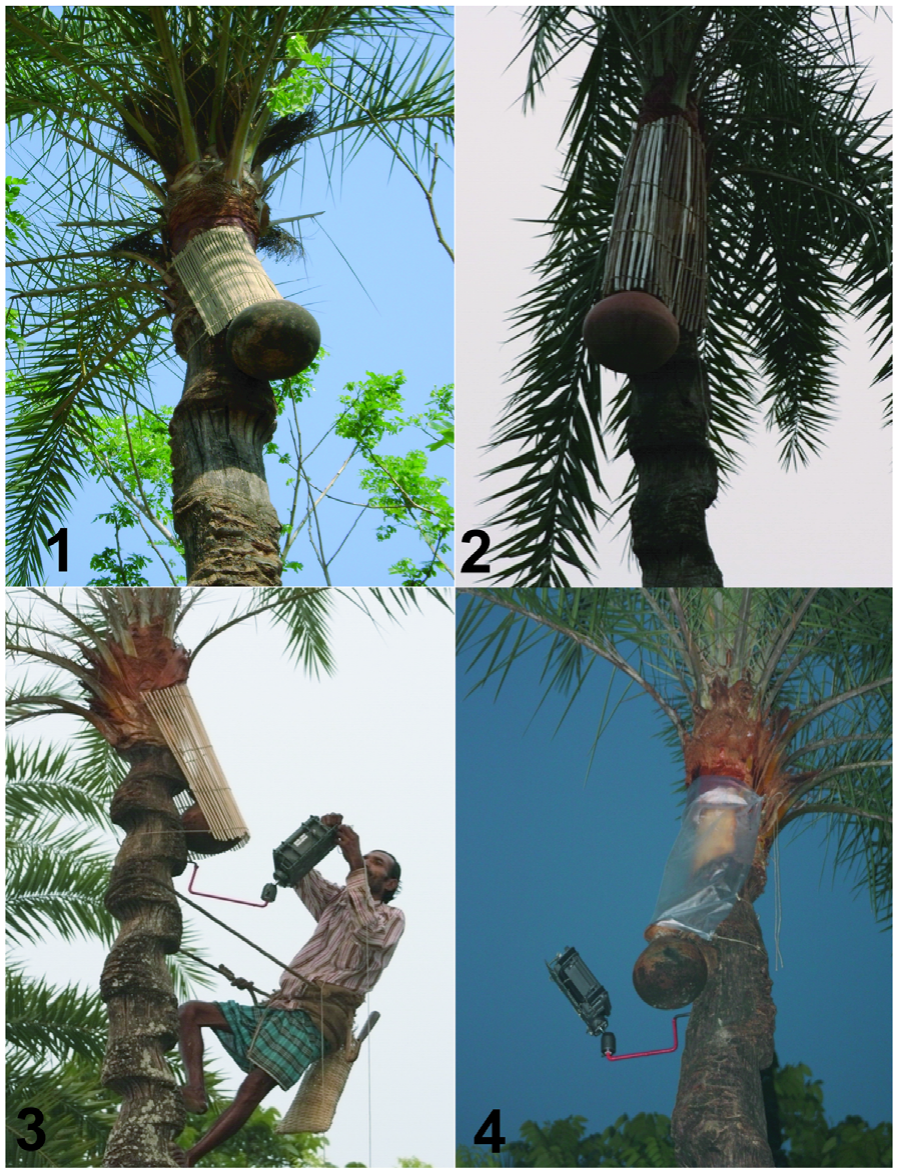
Interventions to prevent bat drinking date palm sap during session two: 1. Bamboo skirt; 2. *Dhoincha* skirt; 3. Jute stick skirt; and 4. Polythene skirt covering the sap producing areas of the date palm tree. In the later pictures, we can see sap harvester setting up an infrared camera.

We conducted two trials in Poromanandopur village (N23° 33′ 49.3″ E89° 41′ 55.2″) in Faridpur district during two sap harvesting seasons: first from January 2009 through February 2009 and we label it as the 2009 data collection period and second was from November 2009 through March 2010, and we label it as the 2010 data collection period. The interventions included bamboo, *dhoincha*, jute stick, and polythene skirts to cover the sap producing surface of the tree [Figure 2]. During 2009, we compared the number of times bats contacting sap to trees with and without bamboo skirt intervention. During 2010, we compared the number of times bats contacting the sap to trees with interventions by bamboo, jute stick, *dhoincha*, and polythene skirts and the bats contacting the sap to trees without any intervention.

### Selecting Date Palm Trees for Intervention During 2009

During 2009 we used a crossover design for applying interventions to the trees. We identified 54 date palm sap producing trees prepared for sap harvesting by a sap harvester in the village. The trees we selected were tall enough so that terrestrial animals, including dogs and foxes, could not reach the sap producing surface and were trees that the harvesters frequently noticed bats visiting. We assigned a unique identification number to each of these trees. Using Microsoft Excel we generated 20 random numbers between 1 and 54 in order to select 20 trees. Each tree was observed for one night with the intervention and for one night without any intervention.

A sap harvester from the study village estimated the average size of the sap collecting area of a date palm tree and made the bamboo skirts that we used for the intervention. Then the harvester climbed the trees and fastened the bamboo skirts over the shaved part, sap stream, tap and opening of the collection pot hung on the trees (Figure 2).

Ten trees were randomly selected to be observed with intervention and 10 were observed without interventions during the first 10 nights of data collection. During the second 10 nights of data collection, the trees were assigned to the opposite group. Every night, we observed two newly shaved date palm trees: one with the bamboo skirt intervention and the other without any intervention so that the observations were matched in terms of time. The date palm sap harvester mounted two infrared cameras (Silent Image^™^ Model RM30 digital cameras (Inclusion of trade names is for identification only and does not imply endorsement by ICDDR,B, by CDC or the Department of Health and Human Services)) on the trees before dusk, one camera per tree. The cameras were triggered by a motion sensor and were focused on the shaved surface, sap stream, tap and the opening of the collection pot. This way, we observed all 20 trees for two nights each; one night with the bamboo skirt applied and one night without the intervention.

### Selecting Date Palm Trees for Intervention and Control During 2010

During 2010, we identified 277 tall date palm sap producing trees prepared for sap harvesting by eight sap harvesters in the same village. From those, we selected 60 trees as controls by the random selection process described above. From the remaining 217 trees, we identified potential matches for the controls by comparing their apparent height, shaving pattern of the trunk of the tree [15]. For every control tree, there was more than one potential match [range: 2–12 trees]. We again entered the tree numbers in Microsoft Excel and generated random numbers to select one tree to receive the skirt interventions for each control. Of the 60 trees selected for intervention, we assigned four interventions: bamboo, *dhoincha*, jute stick and polythene skirts to cover the shaved surface, sap stream, tap, and collection pot (Figure 2) for 15 trees each. Trees for each intervention were assigned by generating random numbers in Microsoft Excel. We followed the same procedures as in 2009 for mounting the cameras and observing a matched pair of trees per night.

During this season, we hired the same sap harvester who worked during 2009 to prepare skirts and mount cameras on trees prepared by 10 different date palm sap harvesters in the village. The length of the shaved surface, sap stream, location of the tap and collection pot varied with the diameter of the trunk of the tree. Accordingly, we made bamboo, *dhoincha*, and jute stick skirts of three different sizes: small (18×22 inches); medium (24×28 inches); and large (30×32 inches). The sap harvester placed one of the three size skirts that entirely covered the shaved surface, sap stream, tap, and collection pot hung on the tree.

### Observation and Data Collection

Details of the observation and data extraction process were described elsewhere [12]. Briefly, an infrared camera was placed for one night per tree from 5∶00 PM to 6∶00 AM; we defined this as a “camera-night” of observation. If there was any movement in the sap collection area the camera took one picture per second for the next five seconds. In the morning, the camera was taken down from the tree and the pictures were transferred to a computer and reviewed.

During the data extraction process we counted all species of fruit bats and defined a “bat visit” as an instance where we could identify a bat flying and/or landing on and around the tree. We defined an event of “bat-sap contact” as an instance of a bat landing, licking (i.e. bat’s tongue contacted the date palm sap), or urinating either on the date palm’s shaved surface, sap stream, tap or collection pot that comes in contact with the sap. We categorized the camera-nights of observations into those with and those without bat-sap contact.

**Figure 3:**
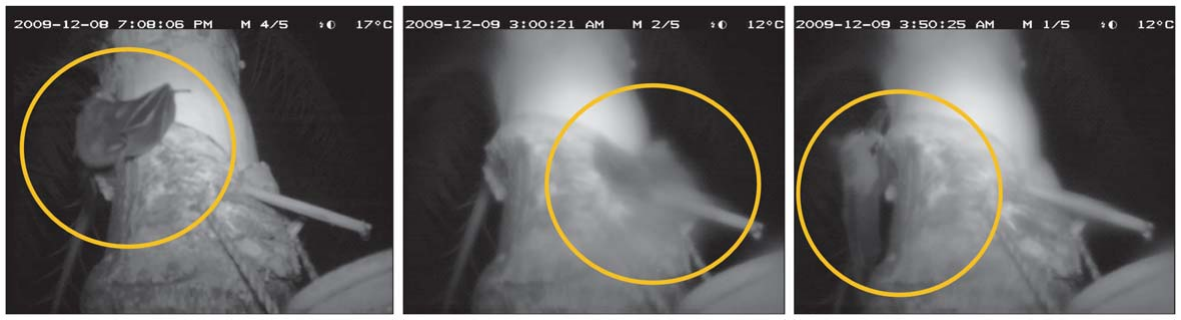
Identifying bats visiting date palm tree on a foggy night: a. 7∶08 PM, no fog, bats are easily identifiable; b. 3∶00 AM, fog blurs visibility but bats are somewhat identifiable; c. 3∶50 AM, fog starts to clear and bats appear clearly in the photos. Each image shows bats in a circle, observation date, time, and ambient temperature when it was taken.

Trained technicians reviewed all of the camera images and recorded the camera data in a structured field data sheet, which included the frequency of bat visits, and their duration and methods of bat-sap contact, and ambient temperature (Figure 3).

Field workers also used a structured questionnaire to ask the sap harvester(s) to assess the volume of sap produced; its appearance (clear or turbid); and the presence or absence of any physical debris originating from the tree itself, insects, and fecal materials of birds, bats or other animals. Field workers asked the sap harvesters how much money he would receive from selling the raw sap for either human consumption or for molasses production.

### Data Analysis

We used descriptive statistics to summarize the frequency of bat visits with and without interventions placed in the trees. We used conditional logistic regression to assess the association between frequency of the events of bat-sap contact with the presence or absence of interventions; and calculated odds ratios. We used a paired t test to assess the difference of the volume of sap production and price with or without interventions. We also used point bi-serial correlation to correlate sap quality (clear or turbid appearance, and presence or absence of debris in sap) with quantity of sap (volume and price) and ambient temperature [17]. We fitted an exponential regression model to explore monthly trends of bat visits in trees where no intervention was used.

### Ethical Considerations

We explained the objectives and the methods of this study to the sap harvesters and the tree owners and obtained informed consent from them before conducting fieldwork. This study was approved by ICDDR,B’s Ethical Review Committee.

## Results

### Date Palm Sap Protection

#### Bamboo skirt intervention in 2009

The number of camera-nights where bats contacted date palm sap (35% versus 85%), p<0.001] and the number of bat-sap contact events (mean 2 versus 32 bat-sap contact per tree per night, p = 0.01) was lower when trees were protected with bamboo skirts compared to when no intervention was used (Table 1).

**Table 1:** Frequencies of bat visits to different parts of date palm sap tree with and without interventions and comparison of sap in terms of appearance, volume and presence or absence of debris.

	Observations in 2009		Observations in 2010				
Types of intervention and control	Bambooskirtn = 20	Withoutskirtn = 20	Bambooskirtn = 15	*Dhoincha*skirtn = 15	Jute stickskirtn = 15	Polytheneskirtn = 15	Withoutskirtn = 60
Mean bat visits per camera night of observation[95% Confidence Interval (CI)]	3 [2.6–4.3]	39 [36–42]	12 [10]–[14]	3 [2]–[4]	8 [7]–[10]	8 [6]–[9]	77 [75–79]
Frequency – landed on the tree	8% (n = 50)	92% (n = 579)	1% (n = 36)	0.2% (n = 8)	1.5% (n = 54)	0.3% (n = 12)	97% (n = 3601)
Bat-sap contact during camera-nightsof observations	35% (n = 7)	85% (n = 17)	0% (n = 0)	0% (n = 0)	7% (n = 1)	0% (n = 0)	83% (n = 50)
Mean bat-sap contact per cameranight [95% CI]	2 [(−0.4) –5]	32 [7–57]	0 [0–0.2]	0 [0–0.2]	1 [0.3–1.3]	0 [0–0.2]	59 [35–84]
**Frequency of bats contacting** **date palm sap [mean (95% CI)]**	**n = 46**	**n = 638**	**n = 0**	**n = 0**	**n = 11**	**n = 0**	**n = 3555**
Shaved surface	29 [26–31]	2 [2]–[3]	0[0–0.2]	0[0–0.2]	1 [0.4–1.3]	0[0–0.2]	51 [49–53]
Sap stream	0 [0–0.2]	3 [2]–[4]	0[0–0.2]	0[0–0.2]	0[0–0.2]	0[0–0.2]	7 [6]–[7]
Tap	0 [0–0.2]	0.05 [−0.05–0.15]	0[0–0.2]	0[0–0.2]	0[0–0.2]	0[0–0.2]	2 [1.5–2]
**Total**	**2 ** [**2]**–[3]	**32 [30–35]**	**0[0–0.2]**	**0[0–0.2]**	**1 [0.4–1.3]**	**0[0–0.2]**	**59 [57–61]**
**Sap characteristics (n = tree-nights** **of observations)**	**n = 20**	**n = 20**	**n = 15**	**n = 16**	**n = 15**	**n = 15**	**n = 60**
Clear appearance of sap (%)	85	85	67	73	67	87	62
Mean volume of sap/night in liters [95% CI]	3.3 [2.5–4.0]	3.1 [2.3–3.9]	3.1 [2.3–3.8]	2.9 [2.0–3.8]	3.6 [2.5–4.8]	2.2 [1.6–2.9]	2.8 [2.3–3.3]
Presence of debris (%)	25	85	80	80	86	60	95

The infrared photography demonstrated that bats were able to access the shaved surface of trees and contact sap when the placement of bamboo skirts did not entirely cover the sap producing surface of the tree. Among the 20 skirts placed on trees, 13 (65%) were wide enough to cover the shaved part and had been properly applied. When these 13 were placed on trees, bats were unable to contact the sap.

#### The skirt interventions in 2010

Out of 60 camera-nights observation at control trees, bats made contact with sap on 50 (83%) nights, with a mean of 50 (SD 94) bat-sap contacts at the shaved part per night. Out of the 45 camera nights observed for three of the interventions, we did not observe any bat contacting sap in trees with bamboo, *dhoincha* or polythene skirts. Smaller non-*Pteropus* bats did contaminate sap with the jute stick skirt one night (7%) when they stuck their tongue through the gap between the sticks of the jute skirt to reach the sap stream (Table 1).

### Frequency of Bat Visits

**Figure 4:**
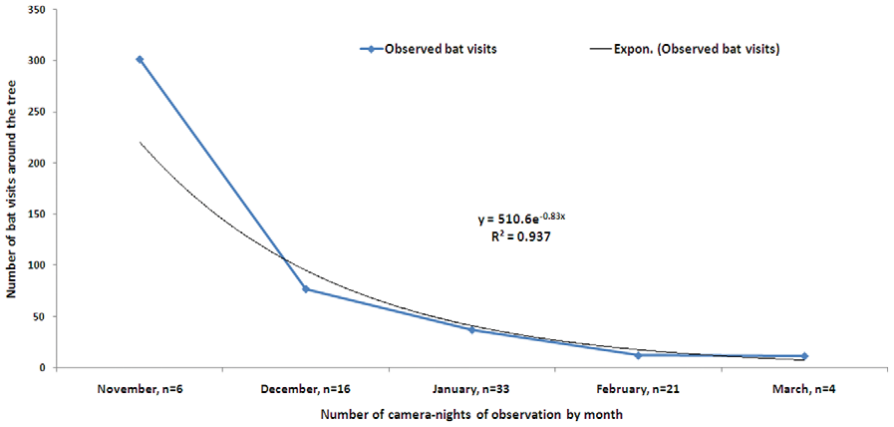
Average number of bat visits per camera-night around the date palm tree (without intervention) during the date palm sap harvesting season in2009 and 2010.

The mean frequency of bat visits to date palm trees without interventions were highest during November, the beginning of sap harvesting season (mean: 242; SD 205 visits) and then decreased in subsequent months reaching the lowest number of visits during March, the end of sap harvesting season (mean: 6; SD 7 visits). In the trees with no intervention, the proportion of bats date palm tree visits decreased 83% each month between November and March (Figure 4).

### Appearance of Sap

During 40 camera-nights of observation in 2009, most (85%, n = 34) of the trees produced clear sap. Date palm trees with and without bamboo skirt were equally likely to produce clear sap [odds ratio (OR): 1; 95% Confidence Interval (CI) 0.2–5.0] (Table 1). During 2010, the trees with intervention were somewhat more likely to produce clear appearing sap than the control trees [OR: 1.7; 95% CI 0.8–3.7], but the difference was not statistically significant. There was no correlation between daily ambient temperature: average (r = 0.04, p = 0.6), minimum (r = 0.01, p = 0.8), or maximum temperature (r = 0.04, p = 0.5) and turbid appearance of sap.

### Presence or Absence of Debris, Volume of Production and Price of Sap

Compared to the trees without intervention, the trees with intervention in both 2009 [OR: 0.06; 95% CI 0.01–0.3] and 2010 [OR: 0.2; 95% CI 0.05–0.6] were less likely to have debris in their sap. The differences were similar for each individual intervention (Table 2). We did not see a significant difference in the volume in sap production between trees with and without interventions in 2009 (*p* = 0.76) and 2010 (*p* = 0.47). Although in the 2009 the harvester reported selling the sap from the trees with interventions at a somewhat higher price than sap that the non-intervention trees (mean price/liter: US$ 0.12 vs. US$ 0.11, p = 0.04), we did not see a significant difference in sap price during 2010, except for the trees with polythene skirt intervention was sold at a higher price (p = 0.05) (Table 2).

**Table 2:** Difference in debris presence, changes in volume of production and price of the sap harvested from date palm trees with and without skirt intervention in the 2009 and 2010 in Bangladesh.

	Volume of sap produced Price of sap Debris in sap						
Interventions and control	Number ofnights ofobservation	Mean volume ofsap in liter/night(Standard error)	95% ConfidenceInterval (CI)of mean	Sap price/liter (USD)(standard error)	95% CIof mean	Odds of havingdebris in sap	95% CI of Odds
***Observations in 2009***							
Bamboo skirts	20	3.9 (0.4)	2–4	0.12* (0.003)	0.12–0.13	0.06	0.01–0.3
No intervention	20	3.1 (0.4)	2–4	0.13 (0.003)	0.12–0.14	Ref	-
***Observations in 2010***							
Bamboo skirts	15	3.1 (0.4)	2–4	0.13 (0.006)	0.11–0.14	0.2	0.03–1.2
*Dhoincha* skirt	15	2.9 (0.4)	2–4	0.13 (0.006)	0.12–0.16	0.2	0.03–1.2
Jute stick skirt	15	3.9 (0.5)	3–5	0.12 (0.009)	0.10–0.15	0.3*	0.05–2.3
Polythene skirt	15	2.6 (0.4)	2–3	0.14* (0.005)	0.12–0.15	0.08	0.01–0.4
No intervention	60	2.9 (0.2)	2–3	0.13 (0.003)	0.12–0.13	Ref	-

* Statistically significant difference at 5% level (two tailed).

## Discussion

This study evaluated interventions that may impede NiV transmission form bats to humans through date palm sap. In almost all cases, bats did not contact sap in trees that were covered with skirt interventions. Although we observed a decrease in debris falling into the sap after applying the interventions, the modest improvements in the appearance of sap, and unit price in sap collected from intervention trees was not significantly different from what was observed from the trees without interventions. Since the interventions do not influence the quality and quantity of the sap that much, there is a need to identify specific motivators, which may encourage the sap harvesters to put skirts on trees and harvest disease risk free sap for human consumption.

During 2009, the width of the shaved part of the tree exceeded the width of the bamboo skirt in a few cases and the bats could gain access to the sap from the left or right side of the shaved surface. The length of the shaved surface, sap stream, and the position of the tap and collection pot varies according to the circumference of the date palm tree. Therefore, skirts of one fixed size may not entirely cover the sap producing surface of a tree and protect the sap from bats. Promoting skirts of three different sizes may provide the tree owners and harvesters with the ability to entirely cover the shaved surface, sap stream, tap, and collection pot of trees with various trunk circumferences. Additionally, the materials used for the interventions showed similar efficiency and the harvesters from different regions may use locally available and convenient material to weave skirts to protect sap.

The event of bat-sap contact during one camera-night of observation with the jute skirt intervention could have been due to the structural characteristics of jute stem [18]. The jute sticks that were used to weave the skirt were not always straight; some of them were curved in the middle. This sometimes created a space between the sticks, through which bats were able to pass their tongue, thereby making contact with the sap. Dhoincha plants also have similar stem characteristics, but we did not observe such bat-sap contact.

The interventions we proposed may prevent physical debris from falling into the sap but the interventions did not influence the clarity or turbidity of sap, or the volume of production. Although several studies reported the volume of sap production and its physical appearance may vary with temperature [19], [20], we did not find any relationship between changes in ambient temperature and production of clear or turbid appearing sap, suggesting that these may be due to the date palm tree’s intrinsic characteristics. Additionally, our observations did not show any significant difference in the volume of sap production after skirt interventions.

We did not see a difference between sap from trees with and without interventions. Before selling the sap to consumers, the harvesters filter their sap with a piece of cloth to get rid of the debris, which may have masked the difference in sap quality produced from trees with and without interventions. However, it remains possible that if the community starts demanding protected sap for drinking, the harvesters may become motivated to exert additional efforts applying interventions and demand more money for their effort.

There was a sharp decline in the frequency of bat visits to the date palm sap producing trees over the sap harvesting season. Bats feed on naturally available food sources throughout the year in Bangladesh [21]. This study identified a large number of bats visiting date palm trees during November-December, which is early in the sap harvesting season. The majority of human Nipah introduction in Bangladesh were identified during January-March [1]. A larger number of bats during the early sap harvesting season of November – January may be attracted by the large volume of sap, easy access to sap producing surface of the tree [12] and high sugar content of the date palm sap [22]. During early sap harvesting season, fewer trees are shaved, which may also influence the higher number of bat visits to each particular date palm tree. Bats shift from one food source to another throughout the year. Since nectar becomes available starting in December in Bangladesh [23], this may reduce bat visits to the date palm trees during that period. We hypothesize that bats may shed NiV throughout the year [24] but humans only get infected when a large enough inoculum of virus contaminates a vehicle that people contact intimately or the virus infects domestic animals. Early in the season when bats frequently feed on date palm sap may be an exceptionally high risk period for spillover of NiV from its wildlife reservoir in *Pteropus* bats to people.

Although observing bat feeding behavior through the infrared camera is a useful method, this is subject to a few limitations, which are described elsewhere [10]. In brief, the cameras may have taken pictures of the same bat visiting multiple times. Therefore, we presented our results as “bat visits” rather than the numbers of bats visiting a tree per night. Despite the sharp focus of the cameras to the shaved surface, in 32 out of 160 observations, heavy fog reduced the visibility for a couple of hours, (Figure 3) and it is possible that we may have missed some bat visits. However, as we observed trees with and without interventions on the same night, the numbers of foggy nights were same for the trees with and without interventions. This study was designed to identify differences in bats feeding behavior and effects of the interventions in the date palm trees. The lack of a significant difference in the quality of sap and price between interventions and controls trees may have resulted from limited statistical power.

Efficient application of bamboo, *dhoincha*, jute stick and polythene skirts by covering the entire shaved part, sap stream, tap and pot effectively protects date palm sap contamination by bats and could prevent NiV transmission from bats to humans. The skirts formed a protective physical barrier and blocked bat contacting the sap [12]. Our findings support the efficacy of the interventions as well as the acceptance of the interventions at community level through pilot studies [15], however, rigorous test of feasibility is required through large scale community based interventions. Future community interventions can promote applying these interventions on date palm trees throughout the sap harvesting season, especially early in the sap harvesting season, to reduce NiV contamination of sap and prevent occasional Nipah spillovers to people.
